# On the Remuneration of Apothecaries

**Published:** 1812-01

**Authors:** 


*To the Editors of the Medical and Physical Journal.*

GENTLEMEN,

MUCH has been written lately respecting medical reform; but the peculiar abuse which has always appeared to me to stand most in need of reform, in the practice of medicine, has scarcely ever been adverted to. I mean the custom of sending medicines to patients in such quantities as to ensure to the practitioner a sufficient reward for his attention and skill. Now I am persuaded, that, if those medical practitioners who are in the habit of looking for their remuneration to the profits arising from the medicines with which they gorge their patients, would but candidly consider the subject, they would agree with me in pronouncing this custom to be not only disgusting to their patients, and extremely disreputable to themselves, but also one of the greatest obstacles to the improvement of the art of medicine.

In consequence of these reflections I have inclosed you the resolutions which the medical practitioners of this town have lately entered into: believing, that, if the practice of medicine were conducted on the principles there stated, it would be, not only more honorable to the practitioners who administer their own medicines, but more agreeable to their patients, and equally conducive to the interests of both.

If you think proper to give this note, together with the resolutions, a place in your Journal, you may leave the prices blank, lest they should appear to the public in the form of an advertisement, which is by no means my intention: but I am so firmly convinced of the purity of the principle on which they are founded, that I candidly confess, I wish all medical practitioners who administer their own medicines, would adopt them. MEDICO-CHIRURGUS.

*Kendal, December* 9, 1811.

Whereas,

WHEREAS, in the town of Kendal, it has not heretofore been usual for the medical practitioners to make any regular charge for their attendance on the sick, but to depend solely for their remuneration on the profits accruing from the medicines administered, which is a method so uncertain and liable to vary, that it generally bears no fair proportion to their trouble and attention:—And whereas, the charges heretofore made for visits out of the town have been but little more than sufficient to defray the costs of horse-hire:—At a meeting, held November 23, 1811, by those medical practitioners whose names are hereunto subscribed,—

It was resolved,

1st.—That it appears to be more conducive to the improvement of the art of medicine, and more honorable for its practitioners, that they should charge for their attendance on the sick, and in proportion to such attendance, than look for remuneration to the profits arising from the medicines administered: as the latter is a custom affording no fair recompense, and which we consider as peculiarly liable to abuse; whilst a charge proportioned to attendance proceeds on the just principle of uniting the interest of the medical attendant with the welfare of his patient.

2d.—That the following be the *lowest charges* made to any patient, excepting servants, laborers, and the poor; as we are convinced, from our experience, that lesser sums would not be a just or adequate recompense to medical practitioners for their attendance and advice.

3d.—That, if the ordinary profits accruing from the medicines administered do not equal the sum of for each visit, such a sum shall be added to the said profits as shall make the whole amount of recompense *not less than* the sum of for each visit in the town.

4th.—That *not less than* the sum of be charged for attending a case of midwifery in the town.

5th.—That *not less than* the sum of be charged by the medical practitioner, in case he be called to visit a patient in the town, between the hours of ten in the evening and six in the morning.

6th.—That *not less than* the sum of per mile be charged for each visit made to a patient out of the town; and that the sum of be added to such charge, in case the practitioner be called between the hours of nine in the evening and six in the morning.

7th.—That *not less than* , together with the sum of per mile, be charged for attending a case of midwifery out of town; but in no case less than

8th.—That these resolutions be printed, and circulated in the town and neighbourhood.

(Signed) Thomas Harrison.
Edward Tatham.
James Towers.
George Forrest.
George Wilson.
Robert Cragg.

